# Dietary carbohydrate source influences molecular fingerprints of the rat faecal microbiota

**DOI:** 10.1186/1471-2180-6-98

**Published:** 2006-11-30

**Authors:** Tine R Licht, Max Hansen, Morten Poulsen, Lars O Dragsted

**Affiliations:** 1 Department of Microbiological Food Safety, Danish Institute for Food and Veterinary Research, Mørkhøj Bygade 19, DK-2860 Søborg, Denmark; 2 Department of Toxicology and Risk Assessment, Danish Institute for Food and Veterinary Research, Mørkhøj Bygade 19, DK-2860 Søborg, Denmark

## Abstract

**Background:**

A study was designed to elucidate effects of selected carbohydrates on composition and activity of the intestinal microbiota. Five groups of eight rats were fed a western type diet containing cornstarch (reference group), sucrose, potato starch, inulin (a long- chained fructan) or oligofructose (a short-chained fructan). Fructans are, opposite sucrose and starches, not digestible by mammalian gut enzymes, but are known to be fermentable by specific bacteria in the large intestine.

**Results:**

Animals fed with diets containing potato starch, or either of the fructans had a significantly (p < 0.05) higher caecal weight and lower caecal pH when compared to the reference group, indicating increased fermentation. Selective cultivation from faeces revealed a higher amount of lactic acid bacteria cultivable on Rogosa agar in these animals. Additionally, the fructan groups had a lower amount of coliform bacteria in faeces. In the inulin and oligofructose groups, higher levels of butyrate and propionate, respectively, were measured.

Principal Component Analysis of profiles of the faecal microbiota obtained by Denaturing Gradient Gel Electrophoresis (DGGE) of PCR amplified bacterial 16S rRNA genes as well as of Reverse Transcriptase-PCR amplified bacterial 16S rRNA resulted in different phylogenetic profiles for each of the five animal groups as revealed by Principal Component Analysis (PCA) of band patterns.

**Conclusion:**

Even though sucrose and cornstarch are both easily digestible and are not expected to reach the large intestine, the DGGE band patterns obtained indicated that these carbohydrates indeed affected the composition of bacteria in the large gut. Also the two fructans resulted in completely different molecular fingerprints of the faecal microbiota, indicating that even though they are chemically similar, different intestinal bacteria ferment them. Comparison of DNA-based and RNA-based profiles suggested that two species within the phylum Bacteroidetes were not abundant in numbers but had a particularly high ribosome content in the animals fed with inulin.

## Background

Prebiotics are dietary carbohydrates, which escape digestion in the small intestine, but undergo bacterial fermentation in the large intestine, and beneficially affect the intestinal microbiota [1]. In addition to a well-established effect on bowel habit and stool bulking, animal studies suggest that ingestion of non-digestible carbohydrates has a protective effect against colon carcinogenesis [2-4].

Inulin-type prebiotics are fermentable fructans that stimulate growth of bifidobacteria, which are regarded as benefical organisms populating the large bowel [5]. Prebiotic products include the long-chained inulin and the short-chained oligofructose, in both of which the monomers are linked by β(2-1) bonds that are not digested in the upper intestinal tract.

Since the cultivable part of the faecal microbiota probably constitutes only 20–50% of the gut microbes [6], it is important to explore effects on this complex ecosystem by use of molecular fingerprinting methods allowing representation of the non-cultivable bacterial species. An example of such a fingerprinting method is Denaturing Gradient Gel Electrophoresis (DGGE) of PCR-amplified 16S rRNA genes, which have proved very useful for analysis of faecal bacteria [7-9]. While the DGGE profiles based on amplified rRNA genes (DNA-DGGE) provides a fingerprint of the composition of the investigated community, they do not necessarily reflect metabolic activities, and could even originate from dormant, lysed or dead cells. The number of ribosomes in prokaryotic cells is correlated to growth rate [10,11], and profiles based on amplified ribosomal RNA sequences (RNA-DGGE) may better reflect the metabolically active bacterial community. Indeed, a recent study showed that alterations of bacterial community profiles after ingestion of prebiotic oligosaccharides by human subjects were only detected in DGGE profiles generated from bacterial rRNA [12].

The objective of the present study was to elucidate the effects of dietary carbohydrates with different digestibility including sucrose, potato starch, inulin, oligofructose and a cornstarch-based control on the composition and activity of the rat intestinal microbiota as measured by physiological parameters, short-chain fatty acid composition, selective cultivation, DNA-DGGE and RNA-DGGE.

## Results

### Weight gain and feed consumption

During the five weeks of feeding, the rats fed the oligofructose containing diet (Table 1) consumed only 79% of the amount of feed consumed by rats in the control group (p < 0.05). Consistently, also the weight gain of these rats was significantly (p < 0.05) lower (80%) than recorded for the control rats. (Data not shown).

### Effects on caecal weight, pH and SCFA content

**Figure 1:**
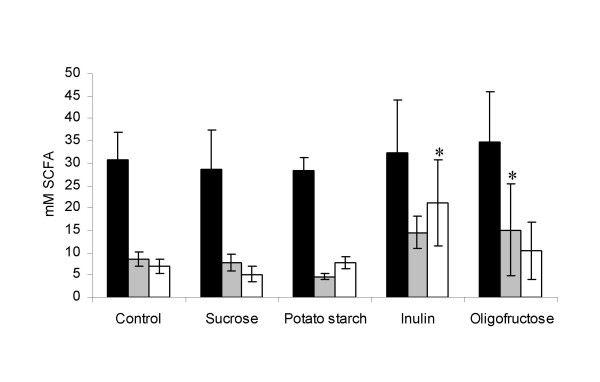
Concentrations of acetic (black), propionic (grey) and butyric (white) acids in caecal contents of rats after 35 days on the respective diets. Averages and standard deviations of each group of 8 animals are presented. Values significantly different from the control group (p < 0.05) are marked by stars.

Caecal weight (with and without contents) and pH of the caecal contents of animals fed either potato starch, inulin or oligofructose were significantly higher (p < 0.05) than in animals fed the control diet (Table 2). Capillary zone electrophoresis revealed a content of acetic acid ranging between 25 and 35 mM in caecum of all animals, while the corresponding concentrations of propionic and butyric acid was generally lower. The concentration of butyric acid and propionic acid in the caecum content of animals fed with either of the fructans was higher than in the other groups. The increase in propionic acid concentration reached statistical significance in animals fed with inulin, whereas the concentration of butyric acid was significantly higher (p < 0.05) in animals given oligofructose (Figure 1).

### Effects on selected cultivable bacterial groups in faeces

**Figure 2:**
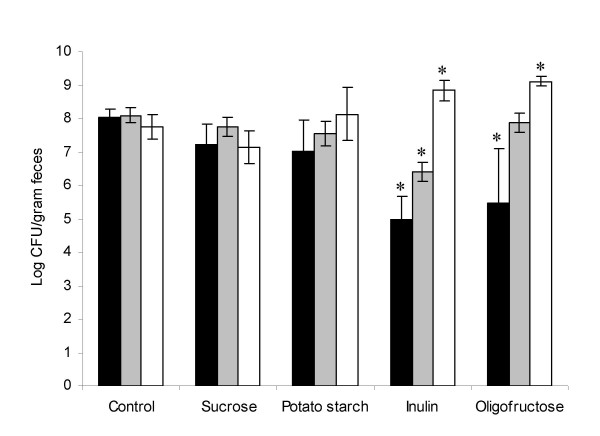
Enumeration of coliform bacteria (black), *Enterococcus *spp. (grey) and lactic acid bacteria growing on Rogosa (white) in faecal samples of rats after 35 days on the respective diets. Averages and standard deviations of four samples, each representing 2 animals, are presented. Values significantly different from the control group (p < 0.05) are marked by stars.

Faecal contents of cultivable aerobic bacteria ranged between 10^7 ^and 10^8 ^per gram, and of cultivable anaerobic bacteria between 10^9 ^and 10^10 ^per gram. Numbers of coliform bacteria were significantly lower, and numbers of lactic acid bacteria growing on Rogosa agar were significantly higher (p < 0.05) in the faeces from animals fed with either of the fructans than observed in the control group (Figure 2). Additionally, numbers of *Enterococcus *spp. were lower in animals on the inulin diet than in the control animals.

### Effects on DGGE fingerprints of faecal microbiota

**Figure 3:**
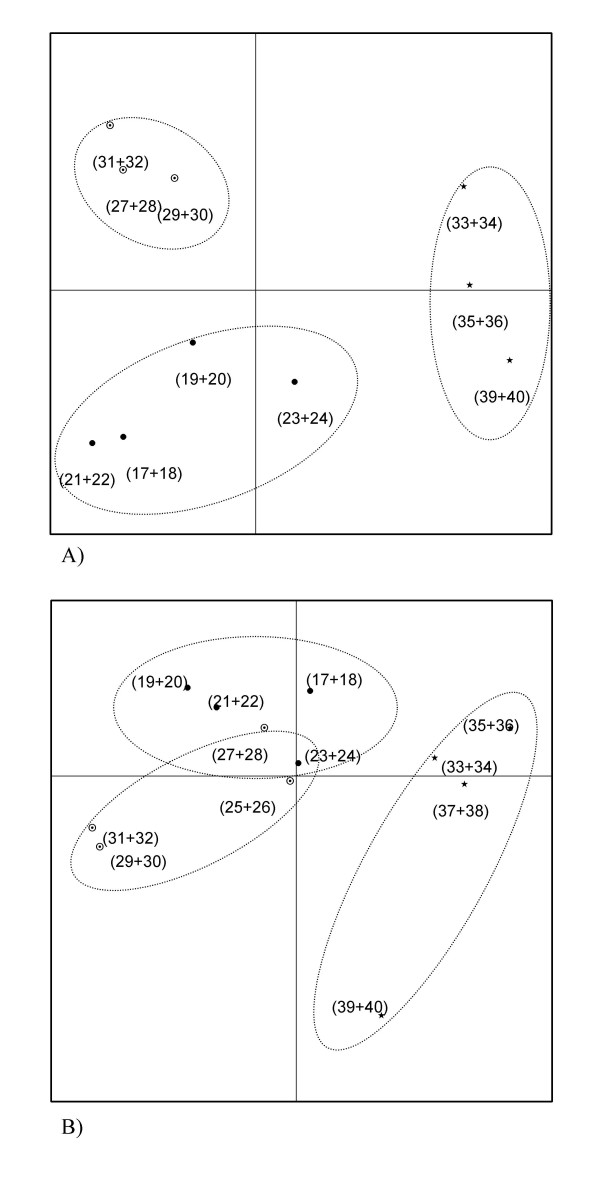
Score plots from Principal Component Analysis of DGGE profiles based on bacterial DNA (A) and RNA (B) extracted from faecal samples. Dotted lines encircles groups fed with control (black dots), sucrose (circles with dots) and potato starch (stars) diets. Animal numbers (Table 1) representing each point are given in parentheses. Two samples were lost during processing of DNA. A) The amount of variability accounted for by Factor 1 is 27.2% and by Factor 2 is 19.4%. B) The amount of variability accounted for by Factor 1 is 26.1% and by Factor 2 is 17.5%.

**Figure 4:**
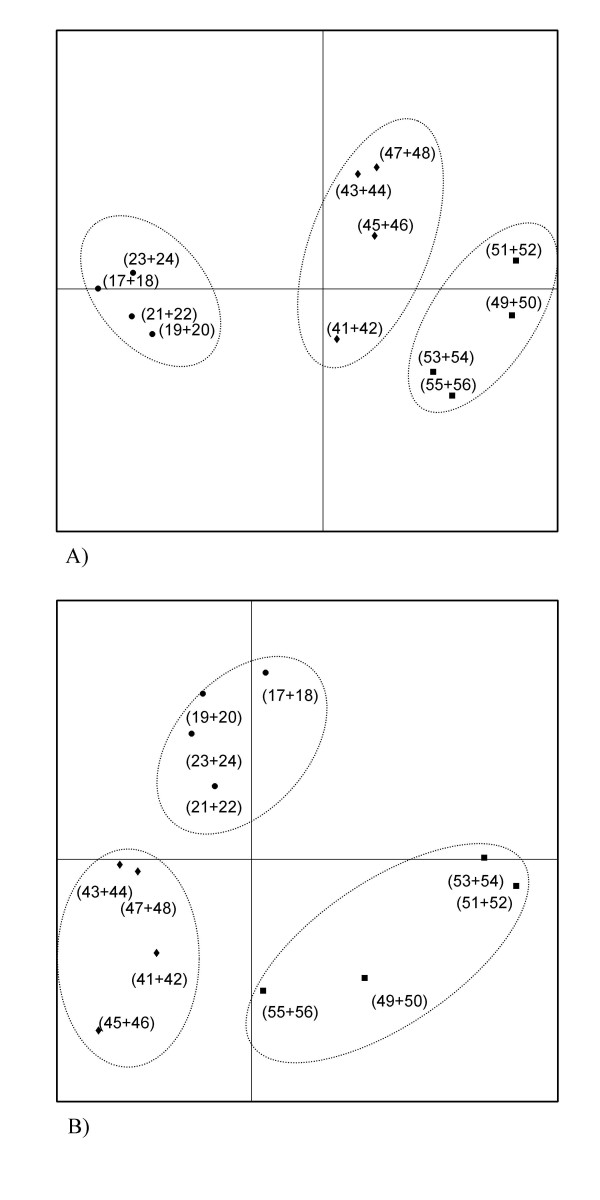
Score plots from Principal Component Analysis of DGGE profiles based on bacterial DNA (A) and RNA (B) extracted from faecal samples. Dotted lines encircles groups fed with control (black dots), inulin (diamonds) and oligofructose (squares) diets. Animal numbers (Table) representing each point are given in parentheses. A) The amount of variability accounted for by Factor 1 is 30.4% and by Factor 2 is 14.1%. B) The amount of variability accounted for by Factor 1 is 19.4% and by Factor 2 is 16.7%

The average number of bands obtained on DNA-based DGGE profiles was 16.5 ± 6, while the corresponding number for RNA-based profiles was 17.4 ± 6. The numbers of bands obtained did not differ significantly between the five dietary groups. Principal Component Analysis of obtained DNA-based as well as RNA-based DGGE profiles revealed that samples from animals belonging to the same dietary group clustered together, but that all dietary groups produced different profiles with the only exception of the RNA-based profiles of the control and sucrose groups, which overlapped (Figure 3 and 4).

**Figure 5:**
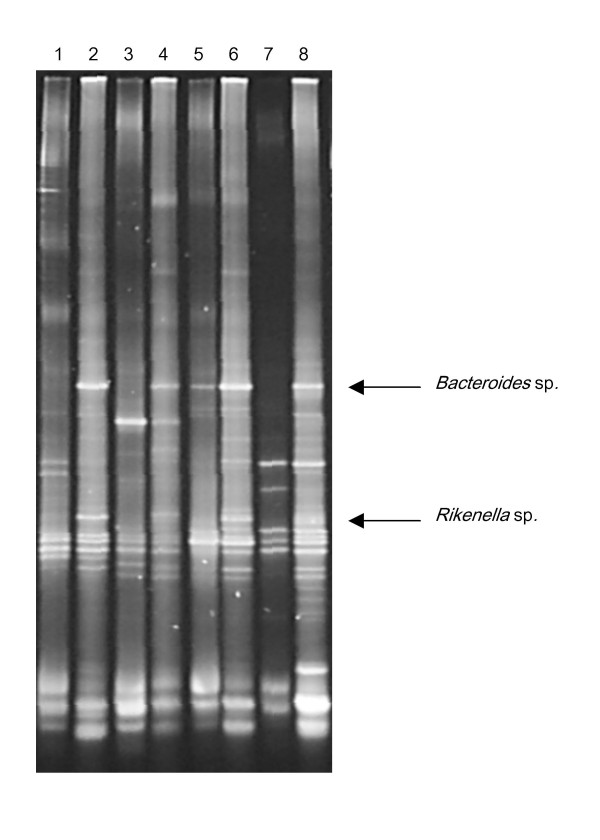
Alternating DGGE profiles based on either extracted DNA (unequal lanes) or rRNA (equal lanes) of faecal samples from rats receiving inulin-based diet. Two bands, which were prominent on all of the rRNA-based profiles, but absent or vague on the corresponding DNA-based profiles were identified as originating from *Bacteriodes *sp. and *Rikenella *sp., respectively.

By comparison by eye of DNA and RNA based profiles obtained from the same faecal samples, two specific bands were observed, which were prominent in the RNA based profiles but absent or vague in the DNA profiles from all animals fed with inulin (Figure 5). A similar evident difference between DNA- and RNA profiles was not observed in samples from any of the other dietary groups (data not shown). Cloning and sequencing of the excised bands resulted in sequence fragments of 173–185 bps, which matched 100% to sequences within the genera of *Bacteroides *and *Rikenella*, respectively, both of which belongs to the order of Bacteroidales (Figure 5).

## Discussion

In order to simulate the food consumption of western consumers, a western-type diet was given to rats in the present study. It is generally recognized that consumption of fructans such as inulin and oligofructose specifically stimulates bifidobacteria, probably due to production of β-fructosidase by these organisms, which makes them able to ferment these substances [13,14]. In the present study, a significant *in vivo *stimulation of species growing on Rogosa agar was observed (Figure 2). This medium is designed to selectively promote growth of lactobacilli, but may give rise to many false positive colonies from faecal samples [15]. Although evidence exists that at least some species of *Lactobacillus *are able to utilize fructans *in vitro *[16], and can be stimulated by these *in vivo *[17], some of the observed increase in colony counts on Rogosa may therefore be attributed to an increase in bifidobacteria [15].

We speculate, that the stimulation of bifidobacteria/lactobacilli may occur also through other mechanisms than specific fermentation of the ingested carbohydrate, e.g. the effect on pH may favour growth of lactic acid bacteria over other gut microorganisms competing for intestinal nutrients.

We recorded increased levels of butyrate and propionate in caecal contents of animals fed with inulin and oligofructose, respectively. Increased butyrate formation is generally considered to be a desirable effect, since butyrate induces apoptosis in cancer cell lines, and is also the preferred fuel for enterocytes [18,19]. It should however be borne in mind that neither lactobacilli nor bifidobacteria produce butyrate, thus the observed increase must result from stimulation of bacterial species other than these.

Our findings of increased caecum weights and decreased caecal pH in animals fed with indigestible fructans (Table 2), indicate increased fermentation in caecum, and are consistent with previously reported studies [20]. Also animals fed with potato starch exhibited increased caecal weight and decreased pH (Table 2) in consistence with a previous study [21]. This indicates that potato starch may have fermentation-stimulating effects on the gut environment.

Analysis of rRNA genes as well as of extracted ribosomal RNA in faeces from animals fed with sucrose, potato starch, inulin, oligofructose and control diets by DGGE resulted in different profiles from animals belonging to each of the five dietary groups, with the only exception of the rRNA-based profiles of the control and sucrose groups, which overlapped (Figure 3 and 4). The average number of bands obtained by DGGE was approximately 17 in all of the dietary groups, indicating that the overall complexity of the microbiota was unaltered by the ingested carbohydrates. Notably, the sucrose-based diet and the cornstarch-based control diet produced different phylogenetic bacterial fingerprints (Figure 3). This indicates, that although cornstarch is not resistant to digestion in the small intestine, a part of it may escape digestion and reach the bacterial community in the large bowel [22]. Cornstarch consists uniquely of glucose subunits, while the subunits of sucrose is glucose and fructose, and it can be speculated that the presence of fructose stimulates gut bacteria other than those stimulated by glucose.

Additionally, it is notable that the phylogenetic composition of faecal bacteria (DNA-based DGGE profiles) as well as the fingerprints of metabolically active organisms in faeces (rRNA-based DGGE profiles) differed markedly between animals fed with the long-chained inulin and the short-chained oligofructose (Figure 4), indicating that the bacterial subspecies specifically stimulated by these two carbohydrate sources are different, although no major differences was observed between these two groups by selective cultivation (Figure 2). Furthermore, inulin caused a significant increase in the level of butyric acid in caecum, while oligofructose caused an increase in the level of propionic acid as compared to the control (Figure 1), which substantiates the indication that different bacterial populations are fermenting these two fructans. Consistently, previous studies revealed different effects of short- and long-chained fructans on large intestine physiology and formation of Aberrant Crypt Foci in rats [21,23]. We speculate that the long-chained fructans travels further into the large bowel before they are degraded than the short-chained fructans, and that this may partly explain that different groups of bacteria are affected. Additionally, when degrading inulin, microorganisms equipped with endo-fructanases will have an advantage compared to organisms producing only exo-fructosidases, while this is less true for oligofructose degradation.

It has been reported that the impact of consumption of oligosaccharides on human faecal microbiota could be detected only by comparison of RNA-DGGE profiles, while no effect on DNA-DGGE profiles was seen [12]. However, a much larger diversity in faecal microbiota from human subjects than in genetically homogeneous experimental animals fed a completely controlled diet is to be expected, and this larger diversity may have masked an eventual effect on the human DNA-DGGE profiles.

Comparison of DNA-based and rRNA-based profiles from the animals fed with inulin revealed that two bacterial species both belonging to the order of Bacteriodales were not sufficiently abundant to be visible on the phylogenetic fingerprint, but had a very high rRNA content, suggesting a highly active protein synthesis, i.e. rapid growth and metabolism (Figure 5). While there has been extensive focus on the specific bifidogenic effect of inulin [13,14], only few reports exist describing effects on other species although it is known that also other intestinal bacteria including species of *Bacteroides *are able to ferment fructo-oligosachharides [24]. Due to its contribution to putrefaction in the intestinal environment, *Bacteroides *is not normally counted among the bacteria which it is desirable to stimulate, although it also contributes to the synthesis of vitamins [14], and has recently been reported to modulate maturation of the host immune system [25]. Therefore, a decrease in the *Bacteroides *population after intake of fructo-oligosaccharides, which has been seen in human microbiota-associated rats [26] and in human volunteers [27], is considered a beneficial effect. Such a decrease is not contradicted by our observations, since the identified species of the phylum Bacteroidetes are not visible on the DNA-DGGE profile (Figure 5), but our current findings suggest that these although they are small, these populations may be very actively growing in the inulin-fed rats. Still, rapid growth would be expected to result in an increase in the population size, and it should be taken into consideration that ribosomes are quite stable, and that the ribosomal concentration in bacterial cells is not down-regulated immediately when growth stops. The explanation for the prominent bands on the RNA-DGGE profiles may therefore be that the high ribosome content in the *Bacteriodetes *species resulted from stimulation of growth of a small subpopulation e.g. associated with a given intestinal compartment. Shedding of bacteria with a high ribosome concentration would occur continuously from such a subpopulation and the stable ribosomes would remain in the bacterial cells excreted with faeces. This has been suggested to be the explanation for the high ribosome content of intestinal *Esherichia coli*, which, similarly, is not correlated to rapid growth of the entire intestinal *E. coli *population [28,29]. Another explanation for the prominent differences between the bands representing the *Bacteroides *and *Rikenella *sp. on the DNA-DGGE and RNA-DGGE gels may be bias of the preceding nucleic acid extraction procedures and/or PCR amplifications. However, since such a difference was observed only in the animals fed inulin, and only for these particular bands, we find it unlikely that it should be the result of PCR bias.

## Conclusion

We conclude that moderate changes in the carbohydrate composition of the feed affected the phylogenetic composition of the intestinal microbiota. In specific cases, also the ribosome content of given species was changed.

This underlines the need for studies, which include phylogenetic analysis of the intestinal microbial communities present as well as assessment of their activities in order to fully explore the effect of prebiotics on the intestinal microbiota.

## Methods

### Animals and housing

Forty 8–10 weeks old male Fisher 344 rats were obtained from Charles River (Sulzfeld, Germany). The animals were housed two by two in standard cages. During the study the temperature was maintained at 22 ± 1°C and relative humidity at 55 ± 5%, air was changed 8–10 times per hour, and fluorescent light was on from 9.00 to 21.00. Diets and acidified water (adjusted to pH 3.05 by citric acid to prevent growth of microorganisms) were provided ad libitum.

Animal experiments were carried out under the supervision of the Danish National Agency for Protection of Experimental Animals.

### Diets and experimental design

The animals were randomized to five different dietary groups with 8 animals per group (Table 1). Dietscontained the same amounts of macro- and micronutrients. Caloric values were balanced, only diets containing inulin and oligofructose had7% less calories than the control diet due to the lower caloric value of these fructans compared to cornstarch.

For one week before these diets were introduced, the animals were all fed with the reference diet. Animals were fed their respective diets for five weeks. Body weight and food consumption were recorded weekly. After euthanization, caecal weight and caecal pH were recorded for each animal, and the composition of short-chain fatty acids (SCFA) in faeces was estimated as described below. Faecal samples excreted during the last 24 hours of the experiment were collected from the cages of the rats, each sample thus representing the two animals, which were caged together.

Inulin (Raftiline^®^) and oligofructose (Raftilose^®^) was purchased from Alsiano (Birkerod, Denmark). Other feed components were obtained from Sigma-Aldrich (Brondby, Denmark), Bie & Berntsen (Rodovre, Denmark), SFK Food (Viborg, Denmark), Unikem (Copenhagen, Denmark), and the Royal Veterinary and Agricultural University (Copenhagen, Denmark).

### Analysis of SCFA composition and pH in caecal samples

Acetic, propionic, and butyric acid in caecal contents were analysed using capillary electrophoresis and indirect UV detection by a method modified from Westergaard et al. [30]. Briefly, ca. 0.1 g of caecum contents were suspended in 9 of alkaline buffer (0.1 M tris, pH 8.7 with 100 μM malonic acid as internal standard), centrifuged (14000 g, 10 min, 4°C) and filtrated using a sterile 0.2 μm filter (Minisart). Samples were kept at -80°C until analysis. Prior to analysis the samples were diluted 60 times by running buffer (0.2 mM 1,2,4-benzenetricarboxylic acid), 8 mM TRIS, 0.3 mM tetradecyltrimethylammonium bromide and 0.15 mM heptakis ((2,3,6-tri-O-methyl)-β-cyclodextrin), pH 7.6). The fused silica capillary (0.75 μm, 80.5 cm and 72 cm to detector window) purchased from Agilent, Germany, was rinsed with 1 M NaOH before each sequence and pre-treated with water for 0.5 min, 0.1 M NaOH for 1 min and runningbuffer for 5 min before each run. Samples were injected by pressure (35 mbar, 2 sec) and run at -30 kV for 12 min on a G1600A ^3D^Capillary electrophoresis Instrument (Hewlett-Packard, Waldbronn, Germany). All chemicals were purchased from Sigma Aldrich, Germany.

### Selective cultivation of bacteria from faecal samples

Anaerobic and aerobic cultivable bacteria in fresh faecal samples were enumerated on Reinforced clostridial agar (Oxoid, Hampshire, England), *Lactobacillus *spp. (and others) on Rogosa (Oxoid), coliforms on MacConkey agar no. 3 (Oxoid) and *Enterococcus *spp. on Slanetz and Bartley agar (Oxoid). Samples were homogenized and appropriate dilutions were plated on the selective agars.

Anaerobes and lactobacilli were incubated anaerobically for 3 days, whereas coliforms, enterococci and total aerobes were incubated aerobically for 1, 2, or 3 days, respectively. All incubations were carried out at 37°C.

### Extraction of bacterial DNA and rRNA from faecal samples

For DNA extraction, frozen faecal samples were diluted 1:10 (w/vol) in phosphate-buffered saline and thawed at 4°C. DNA was extracted from 2 ml of the 10^-1 ^dilution by a method previously described [31], purified using the QIAamp DNA Stool Mini Kit (Qiagen, Hilden, Germany) and stored in 30 μl autoclaved water at -21°C until use.

For RNA extraction, frozen faecal samples were diluted 1:10 (w/vol) in a 1:1 mixture of RNAse-free water and RNALater (Ambion), thawed at 4°C, homogenized and centrifuged at 200 g for 5 minutes. The supernatant was transferred to a new tube and centrifuged at 14.000 g for 5 minutes, and the pellet was resuspended in 1.2 ml of a 1:1 mixture of RNAse-free water and RNALater. The suspension was transferred to a 2 ml Eppendorf tube containing approximately 500 mg of zirconia-silica beads (Biospec Products Inc., Bartlesville, OK, USA). A 30 μl quantity of 10% sodium dodecyl sulphate was added, and the bacteria were lysed by shaking for 4 minutes on a bead beater (Mixer Mill MM 300, Retch, Haan, Germany) at 30 Hz. After centrifugation at 1000 g for 20s, the supernatant was transferred to a new tube and kept at -20°C until RNA isolation by use of Qiagen RNeasy columns (Qiagen) according to the manufacturers instructions. Residual DNA was removed by treatment with DNA-free™ (Ambion). RNA integrity was assessed by electrophoresis of each sample in a 1.2 agarose gel in which 16S and 23S RNA bands were observed.

### PCR and RT-PCR amplification

Aliquots (10 μl) of purified DNA were applied to the following to give a 50 μl PCR reaction mixture: 20 μl of Eppendorf^® ^Mastermix (2.5×) (Eppendorf) and 40 pmol of each of the universal primers HDA1-GC/HDA2 [32] targeting all bacteria. PCR was run in a Peltier Thermal Cycler PTC-225 (MJ Research) as a touchdown PCR. Initial denaturation was at 96° for 5 min, amplification was carried out using 20 cycles including denaturation at 94°C for 1 min, annealing at 65°C for 1 min decreased by 0.5°C for each cycle, and extension at 72°C for 1 min. This was followed by additional 5 cycles of denaturation at 94°C for 1 min, annealing at 55°C for 1 min, extension at 72°C for 1 min, and a final extension at 72°C at 5 min.

Aliquots (10 μl) of purified ribosomal RNA were amplified using the Qiagen OneStep RT-PCR kit (Qiagen) according to the manufacturers instructions, using the same primers as for DNA amplification. 1% (vol/vol) of Rnase Inhibitor (Applied Biosystems) was used in the RT-PCR reactions. Reverse transcription PCR was run in a Peltier Thermal Cycler PTC-225 (MJ Research) with the following amplification program: one cycle consisting of 30 minutes at 50°C (reverse transcription) and 15 minutes at 95°C, followed by 35 cycles of denaturation at 94°C for 30 s, annealing at 56°C for 30 s, and extension at 72°C for 45 s. This was followed by a final extension at 72°C for 10 min. PCR amplification and subsequent DGGE separation was performed twice for each sample.

### Analysis of faecal microbiota by DGGE

DGGE was carried out as previously described [9] using a DCode™ Universal Mutation Detection System instrument and gradient former model 475 according to the manufacturer's instructions (Bio-Rad Labs, Hercules, California). The denaturing gradient was formed with two 9% acrylamide (acrylamide-bis 37.5:1) stock solutions (Bio-Rad) in 1 × TAE (20 mM Tris, 10 mM acetate, 0.5 M EDTA, pH 7.4). The gels were made with denaturing gradients ranging from 25 to 65%. The 100% denaturant solution contained 40% formamide and 7 M urea.13 μl PCR product were mixed with 3 μl loading dye before loading. Gels were run in 1 × TAE at 60°C for 16 hr at 36 V, 28 mA, stained with the fluorescent dye Gelstar (Biowhittaker) for 45 min, and viewed by UV trans illumination. The BioNumerics software, version 3.00 (Applied Maths, Belgium) was used for identification of bands and normalization of band patterns from DGGE gels. Principal Component Analysis of DGGE pattern profiles was performed using the same software. Subtraction of averages over the characters was included in the PCA analysis. Quantitative values (band intensities) were not used.

### Excision, cloning and sequencing of selected bands from DGGE gels

Bands of specific interest were excised from DGGE gels with a sterile razor, placed in 40 μl sterile water, and incubated at 4°C for diffusion of DNA into the water. 33 μl of the sterile water (containing the DNA) was treated with S1 nuclease [12]. The S1 nuclease treated DNA was used in a PCR with HDA1/2 primers without GC-clamp (4 min at 94°C, 20 cycles consisting of 30 sec at 94°C, 30 sec at 56°C, and 1 min at 68°C, and finally 7 min at 68°C). Subsequently the PCR products were directly cloned into pCR^® ^4-TOPO (Invitrogen, UK) according to the manufacturer's instructions, and electroporated into electrocompetent *E. coli *TOP10 cells (Invitrogen, UK) with a single pulse (2500 V, 400 Ω, 25 μF) by use of a Gene Pulser apparatus (Bio-Rad Laboratories, Richmond, CA). Plasmid DNA was isolated from the cells using the Qiagen Mini Spin Prep kit (QIAGEN, Germany), and subjected to PCR (HDA1/2-GC) as earlier described. The PCR product was run on a DGGE gel to check the purity and confirm the melting behavior of the excised band. The inserts were sequenced by MWG (Ebersberg, Germany) using primers T3 and T7. The obtained sequences were compared to known sequences in the Ribosomal Database (RDP, Michigan State University, Release 9.32).

### Statistics

All parameters were tested for normal distribution with the Kolmogorov-Smirnov test used on the residuals. The homogeneity of variance among groups was tested using Levenes test. Log-transformations were performed for data, which did not meet these criteria. Dunnets test was used to compare dosed groups with control where ANOVA indicate a significant single factor effect.

Data not showing normal distribution or homogeneity of variance was analyzed by Kruskall-Wallis test. All statistical calculations were carried out using SAS release 8.02S (SAS Institute Inc., Cary, NC).

## Authors' contributions

TRL carried out the molecular and culture-based microbiological studies, and drafted the manuscript. MH designed the animal studies, and performed the Short-Chain Fatty Acid analysis and the statistics. MP designed the experimental diets and participated in the design and implementation of the animal studies. LOD conceived of the study and participated in its design and coordination. All authors read and approved the final manuscript.

## Figures and Tables

**Table 1:** Composition of Western type diets

Animal no.	17–24	25–32	33–40	41–48	49–56
Western type diets containing (g/kg)	Control^#^	Sucrose	Potato starch	Inulin	Oligofructose

Sucrose	0	150	0	0	0
Potato starch	0	0	150	0	0
Inulin (Raftiline^®^)	0	0	0	150	0
Oligofructose (Raftilose^®^)	0	0	0	0	150
Caseinate	200	200	200	200	200
Cornstarch	592	442	442	442	442
Soy oil	70	70	70	70	70
Corn oil	80	80	80	80	80
Cellulose	12	12	12	12	12
Mineral mixture^a^	32	32	32	32	32
Vitamin mixture^b^	14	14	14	14	14

^a ^Containing in mg/kg diet: 5000 Ca; 3100 P; 3600 K; 300 S; 2500 Na; 1500 Cl; 600 Mg; 34 Fe; 30 Zn; 10 Mn; 0.20 I; 0.15 Mo; 0.15 Se; 2.5 Si; 1.0 Cr; 1.0 F; 0.5 Ni; 0.5 B; 0.1 B; 0.1 V; 0.07 Co.

^b ^Containing in mg/kg diet: 5000 (IU) vitamin A; 1000 (IU) vitamin D_3_; 50 (IU) vitamin E; 5 thiamin; 6 riboflavin; 8 pyridoxol; 2 folic acid; 0.3 D-biotin; 0.03 vitamin B-12; 20 pantothenate; 2600 cholinhydrogentartrat; 400 inositol; 40 nicotinic acid; 1 phylloquinine; 40 p-aminobenzoic acid; 1000 methionine; 2000 L-cystine.

^# ^Serves as reference group.

**Table 2:** Weight and pH of Caecum after 35 days of consumption of Western type diets with the given carbohydrate sources^a^.

	N	(Caecum weight incl. content/bodyweight) × 1000	(Caecum weight excl. content/bodyweight) × 1000	pH of caecum content
Control	8	8.34 ± 3.0	2.35 ± 0.4	6.83 ± 0.3
Sucrose	8	7.23 ± 0.9	1.94 ± 0.1	6.93 ± 0.2
Potato starch	8	16.93 ± 3.8*	3.46 ± 0.9*	6.48 ± 0.1*
Inulin	8	30.48 ± 6.3*	4.83 ± 0.9*	5.64 ± 0.2*
Oligofructose	8	32.27 ± 3.3*	5.54 ± 0.6*	5.75 ± 0.5*

^a^Values are given as Means ± Standard Deviations.

*Values differing significantly (P < 0.05) from the control group.
